# Mutant *KRAS* associated malic enzyme 1 expression is a predictive marker for radiation therapy response in non-small cell lung cancer

**DOI:** 10.1186/s13014-015-0457-x

**Published:** 2015-07-16

**Authors:** Gaurab Chakrabarti

**Affiliations:** Departments of Pharmacology, University of Texas Southwestern Medical Center, 5323 Harry Hines Blvd, Dallas, TX 75390 USA; Departments of Radiation Oncology, University of Texas Southwestern Medical Center, 5323 Harry Hines Blvd, Dallas, TX 75390 USA; Departments of Simmons Comprehensive Cancer Center, University of Texas Southwestern Medical Center, 5323 Harry Hines Blvd, Dallas, TX 75390 USA; University of Texas Southwestern Medical Center, 6001 Forest Park Drive, ND 2.210, Dallas, TX 75390-8807 USA

**Keywords:** Radioresistance, *KRAS*, Glutamine, NSCLC, *ME1*, *GOT1*, ROS

## Abstract

**Background:**

Advanced non-small cell lung cancer (NSCLC) is an aggressive tumor that is treated with a combination of chemotherapy and radiation if the patient is not a candidate for surgery. Predictive biomarkers for response to radiotherapy are lacking in this patient population, making it a non-tailored therapy regimen with unknown outcome. Twenty to 30 % of NSCLC harbor an activating mutation in *KRAS* that may confer radioresistance. We hypothesized that mutant *KRAS* can regulate glutamine metabolism genes in NSCLC and maintain tumor redox balance through transamination reactions that generate cytosolic NADPH via malic enzyme 1 (*ME1*), which may contribute to radioresistance.

**Findings:**

A doxycycline-inducible mouse model of *KRAS*^*G12D*^ driven NSCLC and patient data was analyzed from multiple publicly accessible databases including TCGA, CCLE, NCBI GEO and Project Achilles. *ME1* expression was found to be mutant *KRAS* associated in both a NSCLC mouse model and human NSCLC cancer cell lines. Perturbing glutamine metabolism sensitized mutant *KRAS*, but not wild-type *KRAS* NSCLC cell lines to radiation treatment. NSCLC survival analysis revealed that patients with elevated *ME1* and *GOT1* expression had significantly worse outcomes after radiotherapy, but this was not seen after chemotherapy alone.

**Conclusions:**

*KRAS* driven glutamine metabolism genes, specifically *ME1* and *GOT1* reactions, may be a predictive marker and potential therapeutic target for radiotherapy in NSCLC.

**Electronic supplementary material:**

The online version of this article (doi:10.1186/s13014-015-0457-x) contains supplementary material, which is available to authorized users.

## Background and findings

**Fig. 1:**
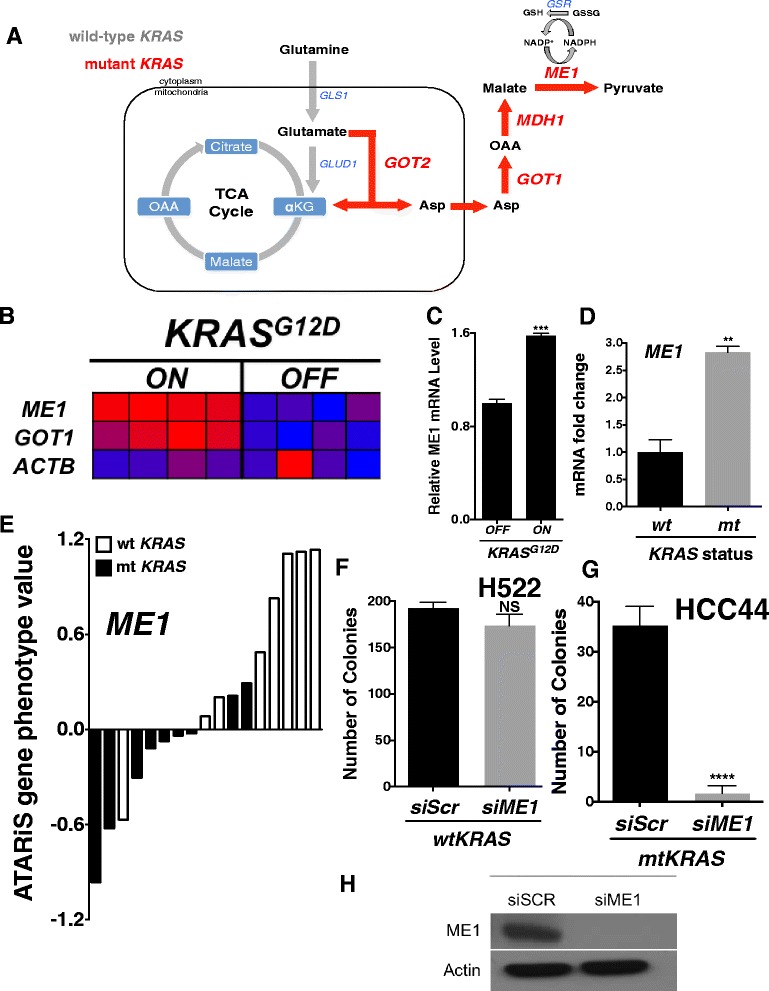
Mutant KRAS is associated with *ME1* and *GOT1* expression in NSCLC. **a** Model of mutant *KRAS*-reprogrammed glutamine utilization (red). GLS1 = glutaminase 1; GLUD1 = glutamate dehydrogenase 1; GOT2 = mitochondrial aspartate aminotransferase; ASP = aspartate; GOT1 = cytosolic aspartate aminotransferase; OAA = oxaloacetate; MDH1 = malate dehydrogenase 1; ME1 = malic enzyme 1; GSR = glutathione disulfide reductase. **b** When fed doxycycline, the mice develop lung tumors that are dependent on constitutive *KRAS* ^*G12D*^ expression [20]. Within 48 h of doxycycline withdrawal, *KRAS* ^*G12D*^ expression was extinguished and whole-genome gene expression analyses of lung tumors were performed. Consistent with mutant *KRAS*-driven reprogramming of glutamine metabolism, *ME1* and *GOT1* levels were up-regulated when *KRAS* ^*G12D*^ was induced *vs* 48 h extinction with doxycycline withdrawl. **c** *KRAS* ^*G12D*^ induction upregulated *ME1* mRNA in mouse doxycycline inducible *KRAS* ^*G12D*^ embryonic fibroblasts derived from the transgenic mice. **d** mRNA expression of *ME1* in mutant *KRAS* vs wild-type *KRAS* NSCLC cell lines. Mutant *KRAS* lines: A549, CALU6, NCIH1155, NCIH1373, NCIH1385, NCIH1573, NCIH2030, NCIH2122, NCIH2347, NCIH460 and NCIH647. Wild-type *KRAS* lines: CALU3, HCC2108, HCC2279, HCC2935, HCC4006, NCIH322, NCIH520, NCIH522, NCIH596, NCIH661 and NCIH838. **e** NSCLC cell line dependencies on ME1 based on ATARiS gene phenotype value assessed from Project Achilles. Black bars = mutant *KRAS* cell. White bars = wild-type *KRAS* cell. Mutant *KRAS* lines: A549, CALU1, CORL23, HCC44, NCIH1650, NCIH1792, NCIH2122, NCIH23 and NCIH441. Wild-type *KRAS* lines: HCC2814, HCC827, NCIH1299, NCIH1437, NCIH1975, NCIH661, NCIH838 and HCC827GR5. **f**-**g** Seven day clonogenic survival assay of H522 and HCC44 with RNAi knockdown of ME1. **h** ME1 western blot in H522; band at 64 kDa. All results were compared using Student’s t-tests as indicated. *p < 0.05; **p < 0.01; ***p < .001

Patients with locally advanced NSCLC that are not candidates for surgery are treated with a combination of chemotherapy and radiation therapy [1]. Clinical trials assessing the efficacy of radiation therapy in this patient population have shown mixed results [2–4]. Furthermore, 20–30 % of all NSCLC harbor an activating mutation in *KRAS* [5]. Interestingly, several studies have demonstrated that the presence of mutant *KRAS* may act as a marker for radioresistance in NSCLC, yet the exact mechanism is not well understood [6–12]. Recent literature has demonstrated that mutant *KRAS* reprograms glutamine metabolism flux in pancreatic cancers through cytosolic aspartate aminotransferase (*GOT1*) and malic enzyme 1 (*ME1*) [13–18]. By synthesizing significant intracellular pools of NADPH via *ME1*, *KRAS*-reprogrammed pancreatic cancers rely on glutamine for redox balance in the face of reactive oxygen species (ROS) production from rapid proliferation and microenvironment stressors (Fig. 1a) [16]. In this context, NADPH is an essential co-factor to blunt ROS formation through the maintenance of intracellular reduced glutathione and thioredoxin [19]. However, to date, there are no studies evaluating whether mutant *KRAS* similarly reprograms glutamine metabolism genes in NSCLC for redox balance and whether this may be a potential mechanism to attenuate ionizing radiation (IR)-induced ROS and DNA damage. Therefore, we characterized glutamine metabolism genes in mutant vs wild-type *KRAS* NSCLC both *in vitro* and *in vivo*, demonstrated the necessity of *ME1* in mutant, but not wild-type, *KRAS* cell lines, and demonstrated that *ME1* gene expression is a predictive marker in the treatment response to radiation therapy in a cohort NSCLC patients.

### Mutant *KRAS* is associated with *ME1* and *GOT1* expression in NSCLC

Gene set enrichment analysis (GSEA) of wild-type vs mutant *KRAS* NSCLC cell lines from the Cancer Cell Line Encyclopedia (CCLE) revealed that genes involved in glutamine dependent redox balance (*ME1* and *GOT1*) were significantly upregulated in mutant *KRAS* cell lines with normalized enrichment scores (NES) >1.48 (Table 1, Additional file 1: Figure S1A).

**Table 1:** GSEA results for mutant *vs.* wild-type *KRAS* NSCLC cell lines

Name	NES	Genes	NOM p-val
NITROGEN_METABOLISM	1.59	GLS	.001
GLUTAMATE_METABOLISM	1.59	GOT1, GOT2, GLS	.001
CARBON_FIXATION	1.48	ME1, ME3, GOT1, GOT2, MDH1	.037

Next, we utilized gene expression data (GSE40606) of a tetracycline operator-regulated Tet-op-*KRAS*^*G12D*^*; p53*^*−/−*^ transgenic mouse model of NSCLC to examine mRNA expression in the KRAS induced (“ON”) and extinguished (“OFF”) states (Fig. 1b). When fed doxycycline, the mice develop lung tumors that are dependent on constitutive *KRAS*^*G12D*^ expression [20]. Within 48 h of doxycycline withdrawal, *KRAS*^*G12D*^ expression was extinguished and whole-genome gene expression analyses of lung tumors were performed. Consistent with our cell line results, *ME1* and *GOT1* levels were significantly upregulated when *KRAS*^*G12D*^ (*n* = 4 mice) was induced *vs* 48 h extinction with doxycycline withdrawal (*n* = 4 mice) (Fig. 1b). We found that *KRAS*^*G12D*^ induction similarly upregulated *ME1* and *GOT1* mRNA in mouse doxycycline inducible *KRAS*^*G12D*^ embryonic fibroblasts derived from the transgenic mice (Fig. 1c, Additional file 1: Figure S1B).

Next, we measured mRNA levels of *ME1* and *GOT1* in 11 mutant and 11 wild-type *KRAS* NSCLC cell lines and found both genes to be significantly upregulated in the mutant cell lines (Fig. 1d, Additional file 1: Figure S1C). Next, to determine if mutant *KRAS* NSCLC cell lines relied on *ME1* for survival, we analyzed 17 NSCLC cell lines from the Project Achilles database, an openly accessible platform of large-scale functional RNAi screens of cancer cell lines to identify genes that affect cell survival [21]. We found that 7 out of 9 mutant *KRAS* cell lines relied on *ME1* for viability, while *ME1* was dispensable in all but one of the wild-type cell lines (Fig. 1e). To verify these results, we knocked down *ME1* (Fig. 1h) in H522, a wild-type *KRAS* line, and in HCC44, a mutant *KRAS* line. Using clonogenic survival assays, we found that *ME1* loss rendered HCC44, but not H522, unable to form visible colonies (Fig. 1f, g). Taken together, our analyses indicate that mutant *KRAS* is associated with *ME1* gene expression in NSCLC and that *ME1* is an essential viability gene in mutant, but not wild-type, *KRAS* cell lines. In support of this observation, *ME1* is a known NRF2 transcriptional target, which itself is positively regulated by mutant *KRAS* signaling via the MAPK pathway [22, 23].

### Targeting glutamine metabolism sensitizes mutant *KRAS* NSCLC cell lines to radiation treatment

**Fig. 2:**
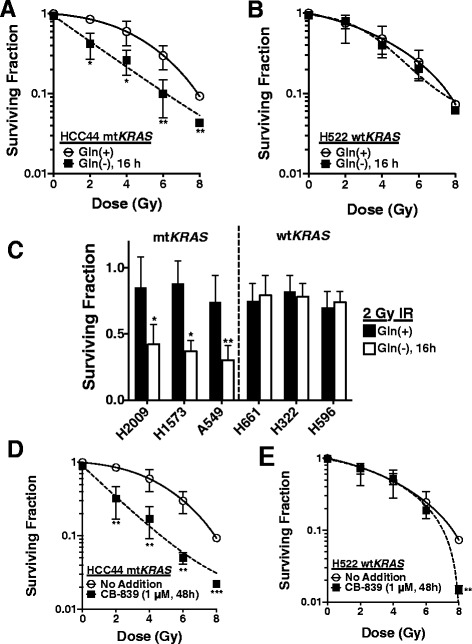
Targeting glutamine metabolism sensitizes mutant *KRAS* NSCLC cell lines to radiation treatment. **a**, **b** Seven day clonogenic survival of HCC44 or H522 after radiation treatment after growth in either complete media or Gln deprived media for 16 h. **c** Clonogenic survival screen of mutant *KRAS* (H2009, H1573 and A549) or wild-type *KRAS* (H661, H322 and H596) NSCLC cell lines grown in either complete media or Gln deprived media for 16 h followed by treatment with 2 Gy of ionizing radiation. **d** Clonogenic survival of HCC44 and H522 pre-treated with 1 μM CB-839 for 48 h followed by treatment with various doses of ionizing radiation. All results were compared using Student’s t-tests as indicated. *p < 0.05; **p < 0.01; ***p < .001

Mutant *KRAS* HCC44 and wild-type *KRAS H522* cells were grown in Gln-free or Gln-containing (2 mM) media for 16 h, then exposed to ionizing radiation and allowed to form colonies for 7 days. Short-term Gln deprivation did not significantly alter clonogenic survival on its own, but did sensitize HCC44 and not H522 cells to radiation, at normally sub-lethal doses (Fig. 2a, b). Using this short term glutamine deprivation protocol, we next screened the mutant *KRAS* NSCLC cell lines H2009, H1573 and A549; and the wild-type *KRAS* NSCLC cell lines H661, H322 and H596 (Fig. 2c). Interestingly, we found that upon glutamine deprivation, mutant, but not wild-type, *KRAS* lines were sensitized to radiation (Fig. 2c). To pharmacologically mimic these results, we pre-treated HCC44 and H522 with the glutaminase 1 (GLS1) inhibitor, CB-839 [24], for 48 h at 1 μM followed by radiation treatment. Consistent with our glutamine deprivation results, HCC44, but not H522, was sensitized to radiation treatment (Fig. 2d).

### *GOT1* and *ME1* expression predicts response to radiation therapy in NSCLC patients

**Fig. 3:**
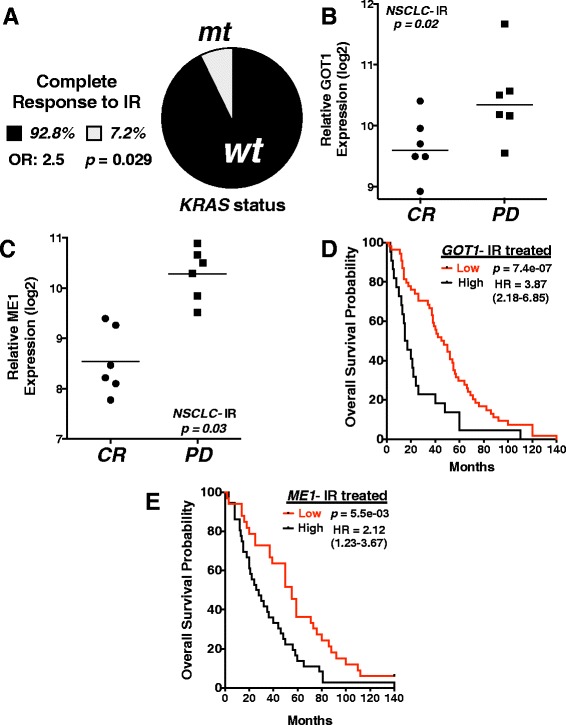
*GOT1* and *ME1* expression predicts response to radiation therapy in NSCLC patients. **a** Percent of complete responders to ionizing radiation (IR) in NSCLC patients separated based on *KRAS* status. Total number of complete responders in TCGA database = 14; wild-type *KRAS* = 13, mutant *KRAS* responders = 1. OR = odds ratio. Results compared using Fisher’s exact test. **b**, **c** *ME1 and GOT1* log2 mRNA expression levels with calculated mean from TCGA NSCLC patients prior to radiation treatment with associated patient outcome after radiation treatment, CR = complete response, disappearance of all target lesions; PD = progressive disease, >20 % increase in the sum of the longest diameter of target lesions. Multiple probes integrated for each gene. **d**, **e** Kaplan-Meier overall survival curves in IR-treated NSCLC patients from KMPLOT database separated into high and low *GOT1* and *ME1* expression. Total number of NSCLC patients analyzed = 73; number of patients with high expression: ME1 = 40, GOT1 = 45; number of patients with low expression: ME1 = 33, GOT1 = 28. All results were compared using Student’s t-tests or a Cox regression analysis unless otherwise stated. *p < 0.05; **p < 0.01; ***p < .001

To expand our *in vivo* and *in vitro* findings into a clinical context, we analyzed mutant *KRAS* status, tumor mRNA expression and RECIST outcomes data from the TCGA in lung adenocarcinoma (LUAD) NSCLC patients who were treated with IR (patient characteristics Additional file 2: Table S1 and Additional file 3: Table S2, https://tcga-data.nci.nih.gov/tcga/tcgaCancerDetails.jsp?diseaseType=LUAD&diseaseName=Lungadenocarcinoma) [25]. Of the 14 LUAD NSCLC patients who had a complete response (CR) to IR treatment, ~93 % (13/14) of the patient’s tumors were wild-type *KRAS*, while only ~7 % (1/14) of the tumors were mutant *KRAS*, suggesting that wild-type *KRAS* tumors may be more radiosensitive compared to mutant *KRAS* tumors, consistent with previous reports (Fig. 3a) [6–12]*. ME1* and *GOT1* expression levels were significantly elevated in those patients who had progressive disease (PD) when treated with IR vs patients who demonstrated a CR after radiation therapy (Fig. 3b, c). Furthermore, we assessed overall survival outcomes in IR treated NSCLC patients (*n* = 73) grouped into high or low *GOT1* and *ME1* expressers. Interestingly, we found that patients with high expression of *GOT1* or *ME1* had significantly worse prognosis over a 140 month time period when compared to low *GOT1* or *ME1* expressers (Fig. 3d, e). Lastly, we did not observe a significant median survival difference between high and low *GOT1/ME1* expressers in NSCLC patients who received chemotherapy, but not IR (Additional file 4: Figure S2A, B). Taken together, this suggests that *ME1* and *GOT1* are predictors to radiation, but not chemotherapeutic, response in NSCLC.

## Conclusions

This multi-database translational study is the first to identify mutant *KRAS* associated glutamine metabolism genes, *GOT1* and *ME1*, as potential radioresistance biomarkers in NSCLC. Our study revealed that elevated expression of *GOT1* or *ME1* is a highly predictive biomarker in radiation treatment, but not chemotherapeutic, outcomes. Additionally, ~93 % of patients with a complete response to IR treatment harbored wild-type *KRAS* in their tumors. To explain these observations, we hypothesize that *KRAS*-reprogrammed glutamine flux through *GOT1* and *ME1* is critical to maintain cytosolic NADPH levels for redox balance and lipid synthesis in NSCLC. In the face of ROS stress, as observed with IR treatment, NADPH is preferentially used to maintain reduced glutathione and thioredoxin 1 to protect cells from ROS damage [19]. In this context, *KRAS* may reprogram NSCLC glutamine metabolism similar to that observed in pancreatic cancer to maintain redox balance, thus providing an oncogene driven mechanism of radioresistance. While there are currently no known specific inhibitors of *ME1* or *GOT1*, targeting upstream glutamine utilization via glutaminase 1 (GLS1, Fig. 1a) inhibition (with BPTES or CB-839) may blunt downstream utilization of glutamine/glutamate through *GOT1* and *ME1*, thus depleting tumor, but not normal tissue, NADPH/GSH production, leading to tumor-specific radiosensitivity while sparing normal tissue [24].

## Materials and methods

### Databases

GSEA of mutant vs wild-type *KRAS* NSCLC cell lines was completed using the Broad Institute’s publically available Cancer Cell Line Encyclopedia (CCLE) (http://www.broadinstitute.org/ccle) [26]. Transgenic mouse data was obtained through GEO Series accession number GSE40606 at Transgenic mouse data was obtained through GEO Series accession number GSE40606. We obtained NSCLC expression, mutation, treatment and outcomes patient data from The Cancer Genome Atlas (TCGA) using the lung adenocarcinoma (LUAD) dataset (https://tcga-data.nci.nih.gov/tcga/tcgaCancerDetails.jsp?diseaseType=LUAD&diseaseName=Lung adenocarcinoma) [25]. Level 2, tumor somatic mutation data was obtained for KRAS for each patient in the analysis (Fig. 3a). Level 2, normalized gene expression data was obtained for GOT1 and ME1 for each patient in the analysis (Fig. 3b, c). Patient characteristics are shown in Additional file 2: Table S1 and Additional file 3: Table S2. Cell line gene dependency data was obtained from Broad Institute’s Project Achilles (http://www.broadinstitute.org/achilles) [21].

### Kaplan-Meier statistics

Survival analysis in radiation treated NSCLC patients (*n* = 73) was conducted using the Kaplan-Meier Plotter webtool (kmplot.com) [27]. Briefly, kmplot segregates each gene into percentile of expression between the lower and upper quartiles and the best performing threshold is used as the final cutoff in a univariate Cox regression analysis. Kaplan-Meier survival plot and the hazard ratio with 95 % confidence intervals and logrank P value is calculated with the Bioconductor package in R.

### Ethical approval and consent

All human data is sourced through The Cancer Genome Atlas (http://cancergenome.nih.gov/), no patients were approached for this study. No consent and no ethical approval were required to utilize this database.

### Survival assay

For clonogenic survival assays, cells were trypsinized and plated onto 6-well plates at 100, 500, or 1000 cells per well in 2 ml of complete media, Gln deprived media for 16 h or complete media containing 1 μM CB-839 for 48 h. Cells were then exposed to IR (at various doses as indicated), allowed to grow for 7 days, washed with PBS and stained with crystal violet solution. Colonies with >50 normal appearing cells were counted and percent survival calculated and graphed with dose.

### RNAi transfection

For siRNA transfection, cells were plated in 10 cm plates at 2 × 10^5^ cells per plate and transfected with either control siRNA or siRNA against ME1 for 48 h followed by clonogenic survival assay.

## Additional files

Additional file 1: Figure S1. (**A**) Raw GSEA data of mutant vs wild-type *KRAS* NSCLC cell lines. Red = overexpressed across all cell lines; blue = under expressed across all cell lines. Absolute top row indicates specific cell lines used in analysis. Gray = mutant *KRAS*; yellow = wild-type *KRAS*. (**B**) *KRAS* ^*G12D*^ induction upregulated *GOT1* mRNA in mouse doxycycline inducible *KRAS* ^*G12D*^ embryonic fibroblasts derived from the transgenic mice. (**C**) mRNA expression of *GOT1* in mutant *KRAS* vs wild-type *KRAS* NSCLC cell lines. Same cell lines as in Fig. 1d. Additional file 2: Table S1. TCGA lung adenocarcinoma patient description. Additional file 3: Table S2.
*GOT1* and *ME1* expression in TCGA lung adenocarcinoma patient tumors with treatment response after IR. Additional file 4: Figure S2. (A, B) Kaplan-Meier overall survival curves in chemotherapy treated NSCLC patients from TCGA database separated into high and low *GOT1* and *ME1* expression. Total number of chemotherapy treated NSCLC patients analyzed = 176; number of patients with high expression: ME1 = 69, GOT1 = 127; number of patients with low expression: ME1 = 107, GOT1 = 49. Logrank p-values not significant.
