# Supplementary figures and images for: Mutant KRAS associated malic enzyme 1 expression is a predictive marker for radiation therapy response in non-small cell lung cancer
Source: Radiat Oncol. 2015 Jul 16;10:145. doi: 10.1186/s13014-015-0457-x (PMC4502640; doi:10.1186/s13014-015-0457-x)

Figure S1

A

Carbon fixation

Glutamate metabolism

Cysteine metabolism

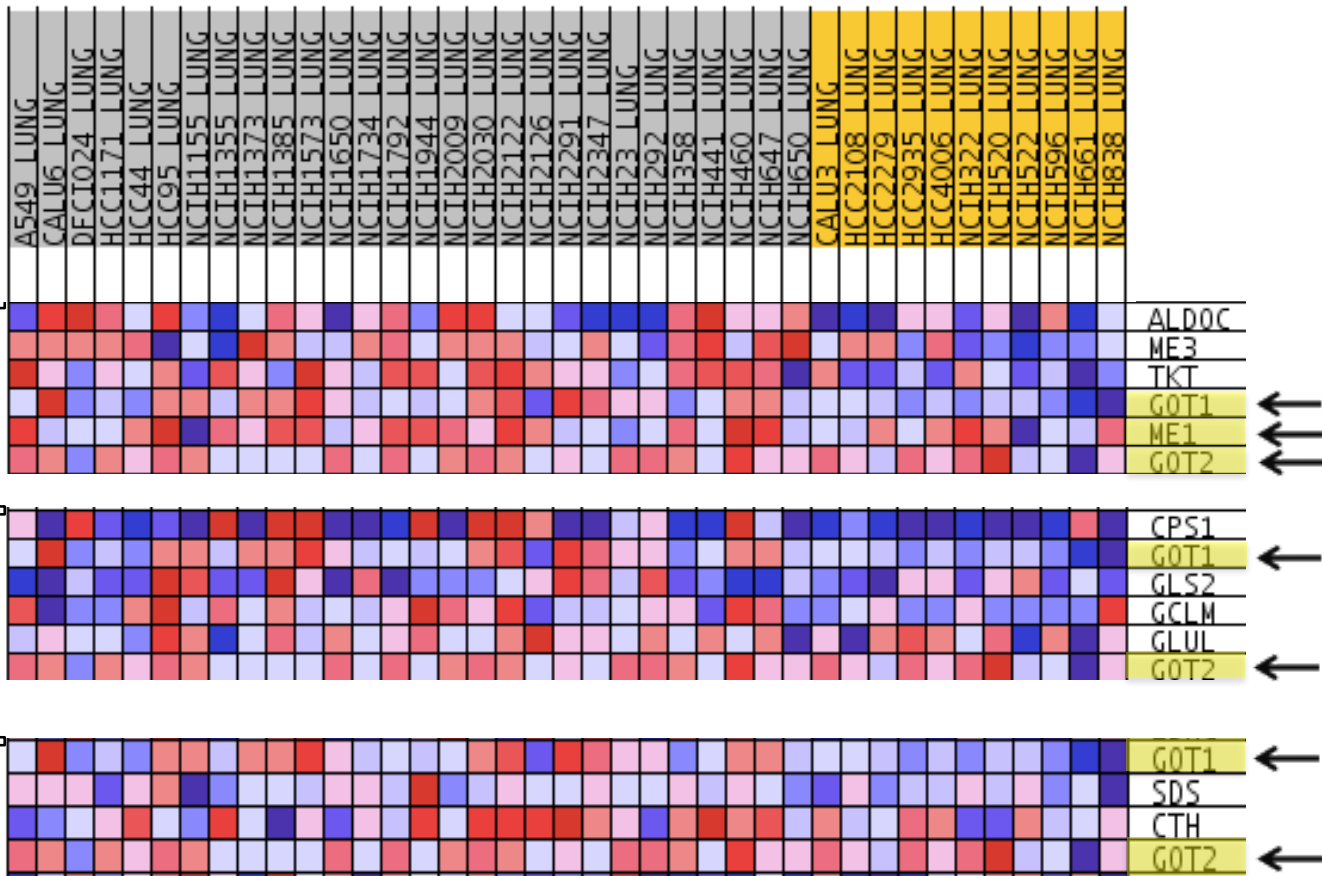

B

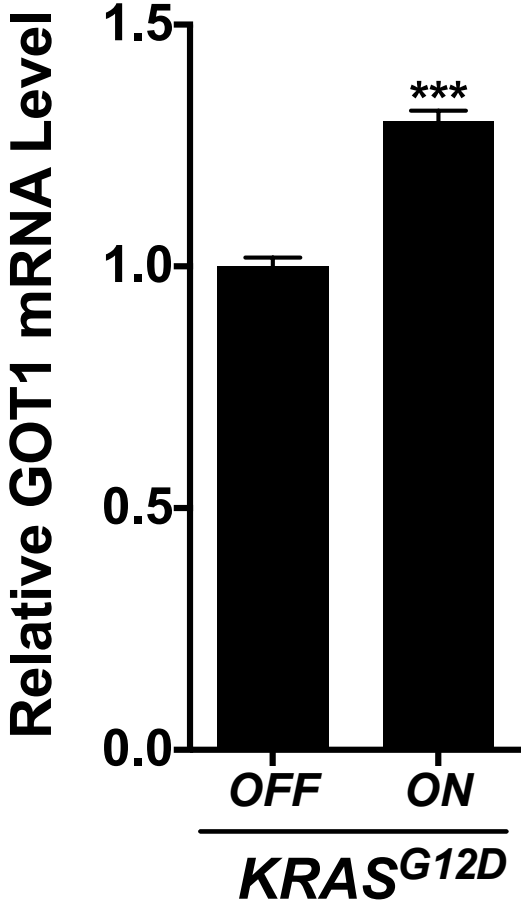

C

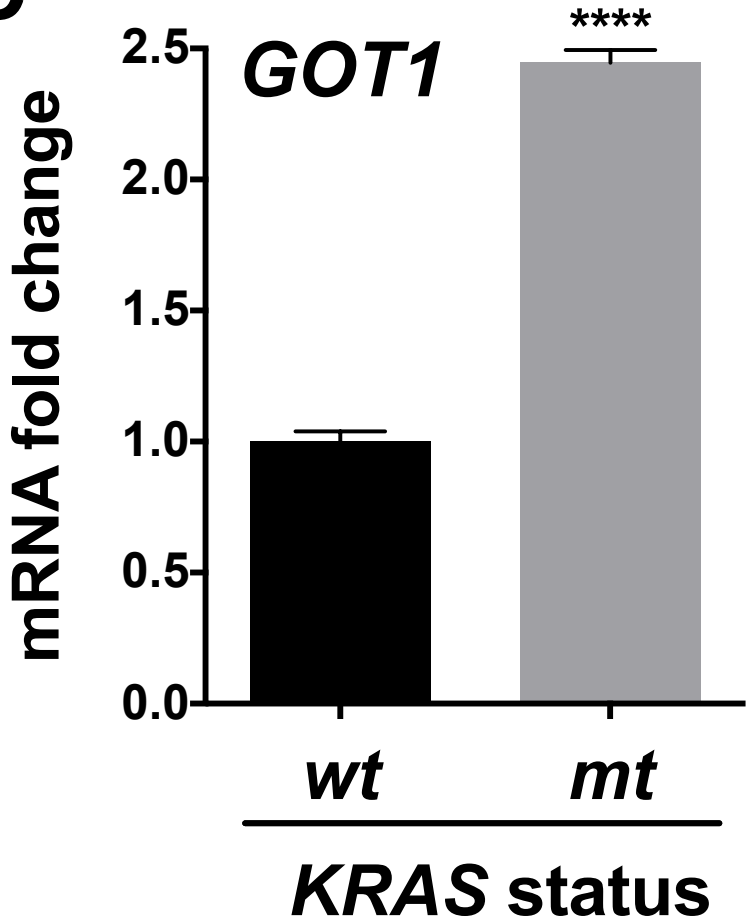

Supplement: Additional file 1: Figure S1. — (A) Raw GSEA data of mutant vs wild-type KRAS NSCLC cell lines. Red = overexpressed across all cell lines; blue = under expressed across all cell lines. Absolute top row indicates specific cell lines used in analysis. Gray = mutant KRAS; yellow = wild-type KRAS. (B) KRAS G12D induction upregulated GOT1 mRNA in mouse doxycycline inducible KRAS G12D embryonic fibroblasts derived from the transgenic mice. (C) mRNA expression of GOT1 in mutant KRAS vs wild-type KRAS NSCLC cell lines. Same cell lines as in Fig. 1d. [file 13014_2015_457_MOESM1_ESM.pdf]

**Figure S2**

**A**

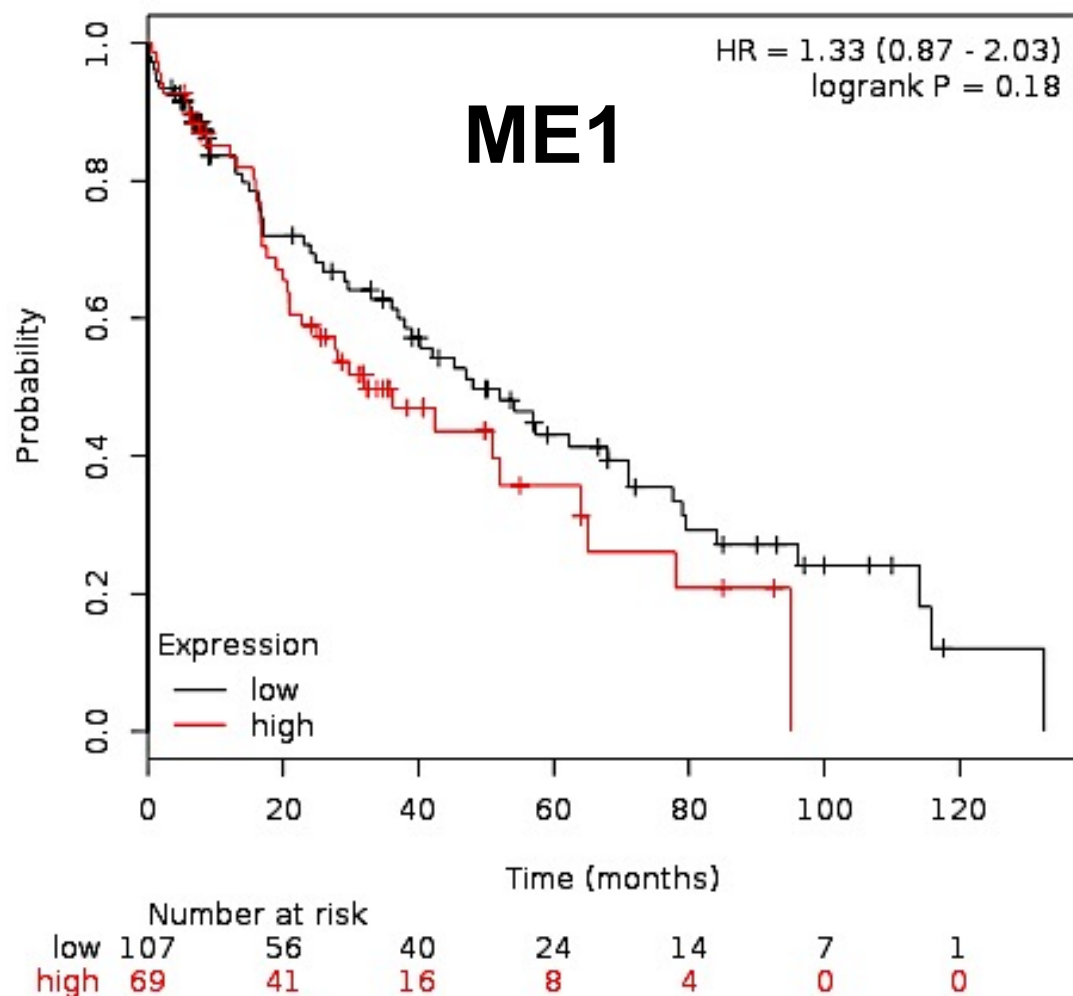

**B**

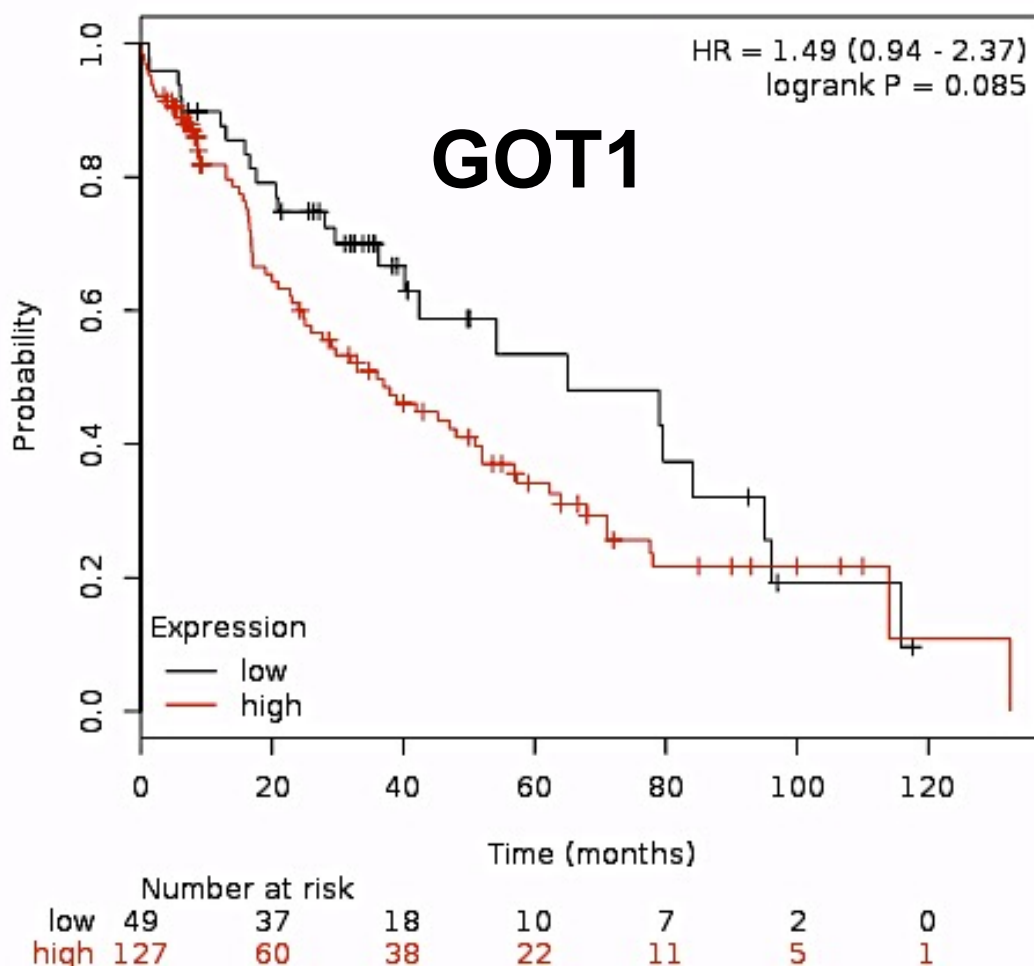

Supplement: Additional file 4: Figure S2. — (A, B) Kaplan-Meier overall survival curves in chemotherapy treated NSCLC patients from TCGA database separated into high and low GOT1 and ME1 expression. Total number of chemotherapy treated NSCLC patients analyzed = 176; number of patients with high expression: ME1 = 69, GOT1 = 127; number of patients with low expression: ME1 = 107, GOT1 = 49. Logrank p-values not significant. [file 13014_2015_457_MOESM4_ESM.pdf]
