# Supplementary material for: Mutant KRAS associated malic enzyme 1 expression is a predictive marker for radiation therapy response in non-small cell lung cancer
Source: Radiat Oncol. 2015 Jul 16;10:145. doi: 10.1186/s13014-015-0457-x (PMC4502640; doi:10.1186/s13014-015-0457-x)
Supplement: Additional file 2: Table S1. — TCGA lung adenocarcinoma patient description. [file 13014_2015_457_MOESM2_ESM.pdf]

Table S1

TCGA lung adenocarcinoma patient description

Patient_Barcode	Complete Response to radiation?	KRAS	Radiation Therapy Type	radiation_therapy_site
TCGA-50-5933	No	*G12C*	EXTERNAL BEAM	Distant Recurrence
TCGA-44-6777	No	*G12C*	EXTERNAL BEAM	Primary Tumor Field
TCGA-44-2668	No	*G12C*	CYBERKNIFE	Distant site
TCGA-73-4659	No	*G12V*	GAMMA KNIFE	Primary Tumor Field
TCGA-64-5774	No	*G12C*	EXTERNAL BEAM	Primary Tumor Field
TCGA-69-7974	**Yes**	*G12C*	EXTERNAL BEAM	Distant site
TCGA-95-A4VK	No	*G12V*	EXTERNAL BEAM	Distant site
TCGA-73-4659	No	*G12V*	Gamma Knife	Distant Recurrence
TCGA-86-7955	**Yes**	WT	EXTERNAL BEAM	Distant Recurrence
TCGA-86-8279	**Yes**	WT	EXTERNAL BEAM	Primary Tumor Field
TCGA-62-8402	**Yes**	WT	EXTERNAL BEAM	Primary Tumor Field
TCGA-78-8660	**Yes**	WT	EXTERNAL BEAM	Primary Tumor Field
TCGA-62-A46Y	**Yes**	WT	EXTERNAL BEAM	Distant site
TCGA-05-4382	**Yes**	WT	EXTERNAL BEAM	Distant site
TCGA-05-5428	**Yes**	WT	EXTERNAL BEAM	Distant site
TCGA-05-5425	**Yes**	WT	EXTERNAL BEAM	Primary Tumor Field
TCGA-55-6968	**Yes**	WT	EXTERNAL BEAM	Primary Tumor Field
TCGA-MP-A4T9	**Yes**	WT	EXTERNAL BEAM	Primary Tumor Field
TCGA-64-5779	**Yes**	WT	EXTERNAL BEAM	Primary Tumor Field
TCGA-91-6847	**Yes**	WT	EXTERNAL BEAM	Regional site
TCGA-05-4424	**Yes**	WT	EXTERNAL BEAM	Distant Recurrence
TCGA-50-5935	No	WT	IMPLANTS	Distant Recurrence
TCGA-50-5942	No	WT	IMPLANTS	Distant site
TCGA-73-4676	No	WT	EXTERNAL BEAM	Primary Tumor Field
TCGA-05-4424	No	WT	EXTERNAL BEAM	Primary Tumor Field
TCGA-44-6774	No	WT	EXTERNAL BEAM	Distant site
TCGA-50-5072	No	WT	EXTERNAL BEAM	Distant site
TCGA-55-6968	No	WT	EXTERNAL BEAM	Distant Recurrence
TCGA-91-6830	No	WT	EXTERNAL BEAM	Distant site
TCGA-91-6847	No	WT	EXTERNAL BEAM	Regional site
TCGA-38-7271	No	WT	EXTERNAL BEAM	Regional site
TCGA-55-7574	No	WT	EXTERNAL BEAM	Regional site
TCGA-78-7145	No	WT	EXTERNAL BEAM	Regional site
TCGA-78-7150	No	WT	EXTERNAL BEAM	Primary Tumor Field
TCGA-78-7152	No	WT	EXTERNAL BEAM	Primary Tumor Field
TCGA-78-7155	No	WT	EXTERNAL BEAM	Regional site
TCGA-78-7220	No	WT	EXTERNAL BEAM	Primary Tumor Field
TCGA-44-7669	No	WT	Cyberknife	Regional site
TCGA-53-7624	No	WT	EXTERNAL BEAM	Primary Tumor Field
TCGA-78-7162	No	WT	EXTERNAL BEAM	Regional site
TCGA-78-7166	No	WT	EXTERNAL BEAM	Local Recurrence
TCGA-78-7536	No	WT	EXTERNAL BEAM	Primary Tumor Field
TCGA-69-7974	No	WT	EXTERNAL BEAM	Primary Tumor Field
TCGA-44-2662	No	WT	EXTERNAL BEAM	Primary Tumor Field
TCGA-64-1680	No	WT	EXTERNAL BEAM	Primary Tumor Field
TCGA-44-3918	No	WT	EXTERNAL BEAM	NA
TCGA-44-4112	No	WT	EXTERNAL BEAM	Local Recurrence
TCGA-38-4630	No	WT	EXTERNAL BEAM	Distant Recurrence
TCGA-49-4507	No	WT	EXTERNAL BEAM	Primary Tumor Field
TCGA-73-4666	No	WT	Gamma Knife	Primary Tumor Field
TCGA-73-4668	No	WT	Gamma Knife	Primary Tumor Field
TCGA-05-5425	No	WT	EXTERNAL BEAM	Primary Tumor Field
TCGA-44-5643	No	WT	IMRT	Primary Tumor Field
TCGA-50-5045	No	WT	Radioactive vicryl Mesh	Distant site
TCGA-50-5049	No	WT	IMPLANTS	Regional site
