# Supplementary material for: Mutant KRAS associated malic enzyme 1 expression is a predictive marker for radiation therapy response in non-small cell lung cancer
Source: Radiat Oncol. 2015 Jul 16;10:145. doi: 10.1186/s13014-015-0457-x (PMC4502640; doi:10.1186/s13014-015-0457-x)
Supplement: Additional file 3: Table S2. — GOT1 and ME1 expression in TCGA lung adenocarcinoma patient tumors with treatment response after IR. [file 13014_2015_457_MOESM3_ESM.pdf]

Table S2

GOT1 and *ME1* expression in TCGA lung adenocarcinoma patient tumors with treatment response after IR

Patient ID	Gender	age	Treatment Response	Status	*GOT1*	*ME1*
TCGA-05-4384	M	66	PD	NA	11.66795491	10.89147615
TCGA-86-7955	M	62	CR	TUMOR FREE	9.957315527	8.465306002
TCGA-86-8279	M	46	CR	TUMOR FREE	9.492075695	8.218630556
TCGA-05-4382	M	68	CR	TUMOR FREE	8.924308363	9.263447149
TCGA-05-5425	M	68	PD	TUMOR FREE	10.18193925	9.120056136
TCGA-05-5428	M	57	CR	TUMOR FREE	9.849773039	10.96241295
TCGA-05-4424	M	70	PR	WITH TUMOR	10.34071783	10.65979784
TCGA-55-6968	M	61	PD	WITH TUMOR	10.56730367	9.84414929
TCGA-53-7624	F	40	PD	WITH TUMOR	10.50298462	10.50211117
TCGA-62-8402	F	73	CR	WITH TUMOR	10.60442035	7.776456063
TCGA-78-8660	M	69	PD	WITH TUMOR	10.16245062	10.29830342
TCGA-MP-A4T9	F	54	CR	WITH TUMOR	9.702757439	8.101167851
